# Linking late Paleoindian stone tool technologies and populations in North, Central and South America

**DOI:** 10.1371/journal.pone.0219812

**Published:** 2019-07-18

**Authors:** Keith M. Prufer, Asia V. Alsgaard, Mark Robinson, Clayton R. Meredith, Brendan J. Culleton, Timothy Dennehy, Shelby Magee, Bruce B. Huckell, W. James Stemp, Jaime J. Awe, Jose M. Capriles, Douglas J. Kennett

**Affiliations:** 1 Department of Anthropology, University of New Mexico, Albuquerque, New Mexico United States of America; 2 Center for Stable Isotopes, University of New Mexico, Albuquerque, New Mexico, United States of America; 3 Department of Archaeology, Exeter University, Exeter, United Kingdom; 4 Institute of Energy and the Environment, Pennsylvania State University, University Park, Pennsylvania, United States of America; 5 School of Human Evolution, Arizona State University, Tempe, Arizona, United States of America; 6 SWCA Environmental Consultants, Carlsbad, New Mexico, United States of America; 7 Department of Sociology, Anthropology and Criminology, Keene State College, Keene, New Hampshire, United States of America; 8 Department of Anthropology, Northern Arizona University, Flagstaff, Arizona, United States of America; 9 Department of Anthropology, Pennsylvania State University, University Park, Pennsylvania, United States of America; 10 Department of Anthropology, University of California at Santa Barbara, Santa Barbara, California, United States of America; Max Planck Institute for the Science of Human History, GERMANY

## Abstract

From the perspective of Central and South America, the peopling of the New World was a complex process lasting thousands of years and involving multiple waves of Pleistocene and early Holocene period immigrants entering into the neotropics. These Paleoindian colonists initially brought with them technologies developed for adaptation to environments and resources found in North America. As the ice age ended across the New World people adapted more generalized stone tools to exploit changing environments and resources. In the neotropics these changes would have been pronounced as patchy forests and grasslands gave way to broadleaf tropical forests. We document a late Pleistocene/early Holocene stone tool tradition from Belize, located in southern Mesoamerica. This represents the first endogenous Paleoindian stone tool technocomplex recovered from well dated stratigraphic contexts for Mesoamerica. Previously designated Lowe, these artifacts share multiple features with contemporary North and South American Paleoindian tool types. Once hafted, these bifaces appear to have served multiple functions for cutting, hooking, thrusting, or throwing. The tools were developed at a time of technological regionalization reflecting the diverse demands of a period of pronounced environmental change and population movement. Combined stratigraphic, technological, and population paleogenetic data suggests that there were strong ties between lowland neotropic regions at the onset of the Holocene.

## Introduction

Lack of knowledge of the Paleoindian period in southern Mesoamerica, a critical early migration bottleneck, has impeded our understanding of the peopling of the Americas and how early New World migrants adapted to emergent tropical environments. Here we present new archaeological and chronological data from stratigraphic excavations in unusually well preserved rockshelter contexts in southern Belize. We securely reassign the chronology of a stone tool technocomplex to 12,000–9,300 years ago linking it to changes in stone tool technology in North America and tropical Central and South America. This is the first securely dated Paleoindian tool technocomplex for southern Mesoamerica.

Our data indicate the late Paleoindian period is characterized by movement away from technological uniformity towards increasing diversification and the establishment of regional traditions and support genetic evidence of strong relationships between Central and South America during the Paleoindian period. Prior to 13,000 calendar years before the present (BP), bands of ice age humans migrating southward from the temperate forests and plains of western North America (NA) crossed the bottleneck of the Isthmus of Tehuantepec to arrive in southern Mesoamerica [1]. Those pioneers encountered new plant and animal species of the neotropic biota [2,3] in mosaic landscapes of semi-tropical gallery forests and mixed patchy grass and scrublands [4,5]. Their arrival coincided with the onset of climatic and environmental changes brought about by the end of the ice age, when warmer and wetter conditions [6,7] drove the emergence of broadleaf tropical rainforests with high biological diversity. Across the Americas, these Holocene environmental changes demanded ecological learning [8] and in the neotropics the adaptation of toolkits developed during the cooler and drier Pleistocene to warmer wetter conditions. One component of these toolkits was flaked stone tools, which are cultural products that are consistently preserved. Generally made of sedimentary rock composed of microcrystalline or cryptocrystalline quartz called chert or sometimes of volcanic obsidian, these tools have been an essential line of evidence regarding the dispersal of humans in the Americas, the transmission of cultural knowledge, and human responses to changing environments. In both NA and South America (SA) archaeological specialists have used changes in bifacial stone tools as one indicator of human adaptation to ecological diversity, linking technological features to regionally distinct cultural responses and food sources and exchange networks [9–14].

**Fig 1:**
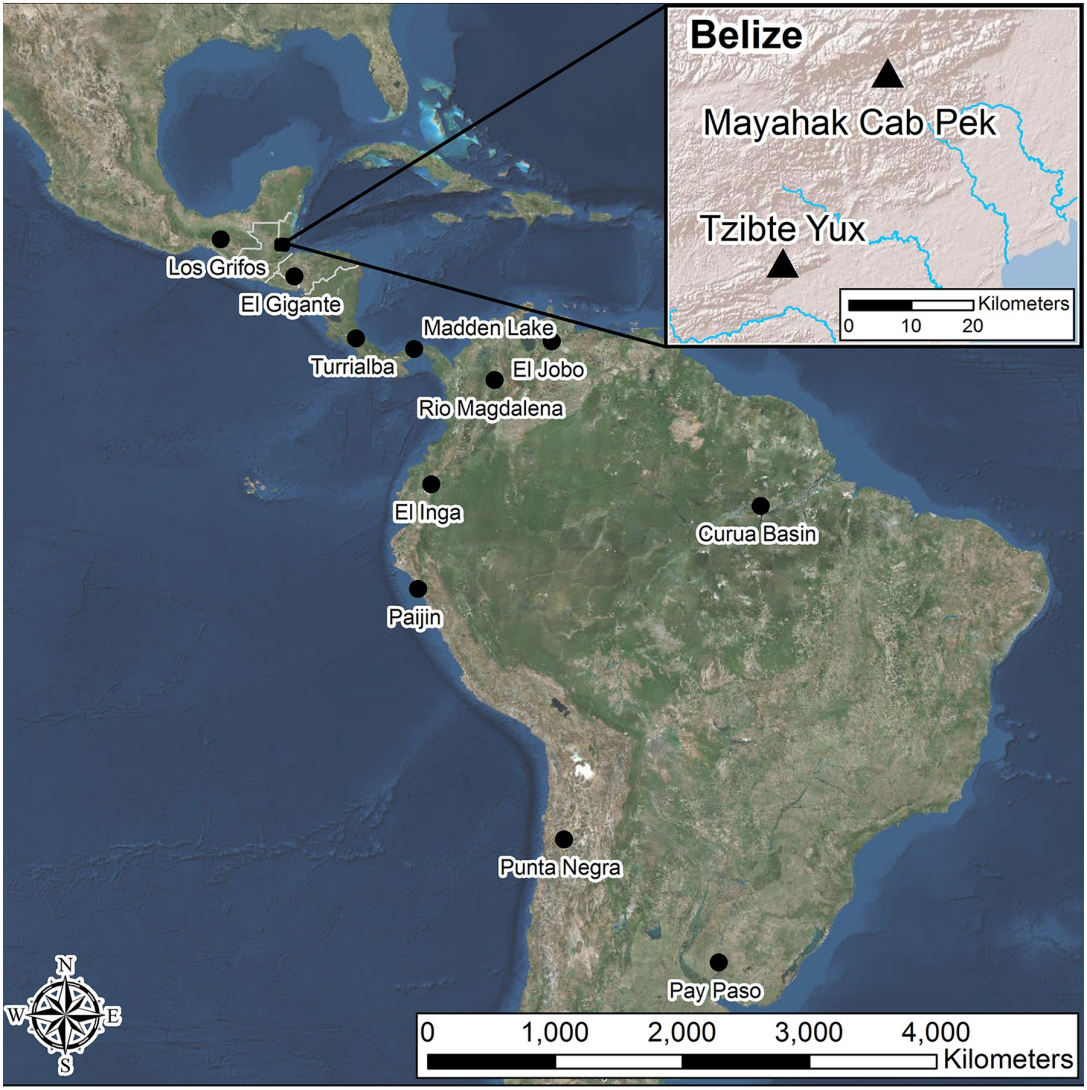
Regional map. This relief map shows southern Mesoamerican and South American sites discussed in the text. The inset box shows a relief map of southern Belize with our study area and the rockshelter sites Tzibte Yux and Mayahak Cab Pek. Base map images are the intellectual property of Esri and is used herein under license. Copyright 2018 Esri and its licensors. All rights reserved.

During archaeological excavations we recovered what we show below to be temporally diagnostic late Paleoindian bifacial tools. They come from stratified contexts in two well-dated rockshelters in southern Belize (Fig 1) where we also recovered numerous expediently made modified flakes and some formal chert tools that likely were used for cutting or scraping, as well as hundreds of simple chopping, hammering, and grinding tools made from locally available river cobbles. Based on radiocarbon dates and Bayesian depositional models [15,16] the minimum ages of these stone tools are 12,000–9,300 CalBP (calibrated radiocarbon years before 1,950 CE). The distinctive form of these large, straight stemmed, barbed bifaces with flat to slightly concave bases and frequently featuring unifacial beveling on alternate edges, classifies them typologically as Lowe points, which were previously assigned [17] to the Late Archaic 4,500–3,900 BP (years before the present based on phases or chronological approximation). These sites represent the first stratified contexts for Lowe bifacial tools and the first designation of a well-dated lithic type to a Paleoindian technocomplex native to southern Mesoamerica. We argue that our revised chronology for Lowe fits a general trend in NA and SA towards diversification of stemmed and barbed bifaces by ~12,000 BP, coinciding with the dramatic climatic and environmental changes at the start of the Holocene [18,19]. Lowe and several related types show affinity with tools produced in lower Central America (CA) and Amazonian SA, as well as links with some NA technocomplexes. The Lowe bifaces from this southern Central America study area bear no relationship to Lowe Flared Base points that date to the late Holocene in the North American Midwest [20]. Our findings are consistent with analysis [1] of ancient DNA that indicates strong genetic relatedness between late Paleoindians in Belize and modern and ancient populations in lower CA and SA following waves of migration from NA. Thus, our data have implications for understanding both Paleoindian and Archaic people in southern Mesoamerica and their relationships with populations in both NA and SA.

### Paleoindian chronologies in southern Mesoamerica

In southern Mesoamerica even rudimentary absolute chronologies dating Paleoindian and Archaic period stone tools are lacking. This stands in contrast to the much later Classic Period Maya where chronologies have been refined over the past century based on radiocarbon dates and hieroglyphic texts [21]. With rare exceptions [22,23] all suspected examples of early bifacial tools in southern Mesoamerica come from undated contexts, primarily surface finds or as poorly documented discoveries. Lacking secure contexts, reconstructions of the earliest periods of human activity in southern Mesoamerica have relied primarily on the comparison of stone tools to those from other regions. The result has been significant uncertainty in timing of important phase-changes that mark shifts in cultural practices. These include the transition from the early to late Paleoindian periods, likely reflecting increasing regionalization as populations adapt to incipient Holocene climate conditions and localized foraging strategies, or the change from the Late Paleoindian to the Archaic periods, reflecting increasing management of plant resources for subsistence and other economic purposes. In southern Mesoamerica interpretation of the early phases of human occupation are largely constrained by comparison to shifts in human-environmental interaction and social organization in NA, rather than neotropical regions of CA. This is entirely due to a lack of well dated late Pleistocene and early Holocene archaeological sites in the region.

Based on comparisons to NA and SA, the oldest bifacial tools found in Central America (CA) are basally thinned lanceolate and fluted Clovis projectile points [22,24]. The latter often have concave bases and constricted midsections referred to as “waisted Clovis” [25,26], after a Clovis variety found in the southeastern US [27]. In SA one of the most widespread and best dated Paleoindian tool types is the stemmed Fishtail Projectile Points (FPP) [28]. While examples of Clovis, waisted Clovis, and FPP have been recovered from southern Mexico and CA, associated dates are only available for two sites. At Los Grifos rockshelter in Chiapas a waisted Clovis and a FPP were found in contexts dating to 10,378–9,555 calBP, and at Los Tapiales, an open air site in highland Guatemala, a fluted stem base was recovered from shallow excavations in contexts bracketed between 13,399–9,561 calBP [29,30]. Revised dates for NA Clovis that range from 13,250–12,800 calBP [31] to perhaps as early as 13,500 CalBP [32,33] and SA FPP from 12,900 to 11,500 calBP [34,35] indicate less than two centuries of overlap, placing SA on a separate trajectory from NA in developing lithic traditions. In western NA, Clovis is followed closely by the also short-lived (12,610 to 12,170 calBP) Folsom biface tradition [36], examples of which are not found in southern Mesoamerica or CA. Following these traditions, both NA [37] and SA [38–40] late Paleoindian stone tools show increasing diversity.

In southern Mesoamerica one of the earliest attempts to develop a Paleoindian and Archaic lithic chronology was the Belize Archaic Archaeological Reconnaissance (BAAR), directed by R.S. MacNeish, an iconic figure in New World archaeology. BAAR assigned undated examples of what we call the Lowe complex to their Lowe-Ha (11,000–9,500 BP) and Sand Hill (9,500–8,000 BP) phases, with some examples labeled as “Pedernales-like”, “Madden Lake-like”, and “Bulverde-like” [41–43], in reference to names of lithic types in NA and a well-known but undated Paleoindian locale in Panama [44]. The BAAR bifaces were later reassigned as types rather than phases [45] and designated as stemmed FPP and lanceolate points “resembling” Plainview (for Lowe-Ha) and La Mina and Pedernales (for Sand Hill).

### Reassessing the chronology of the Lowe complex

**Fig 2:**
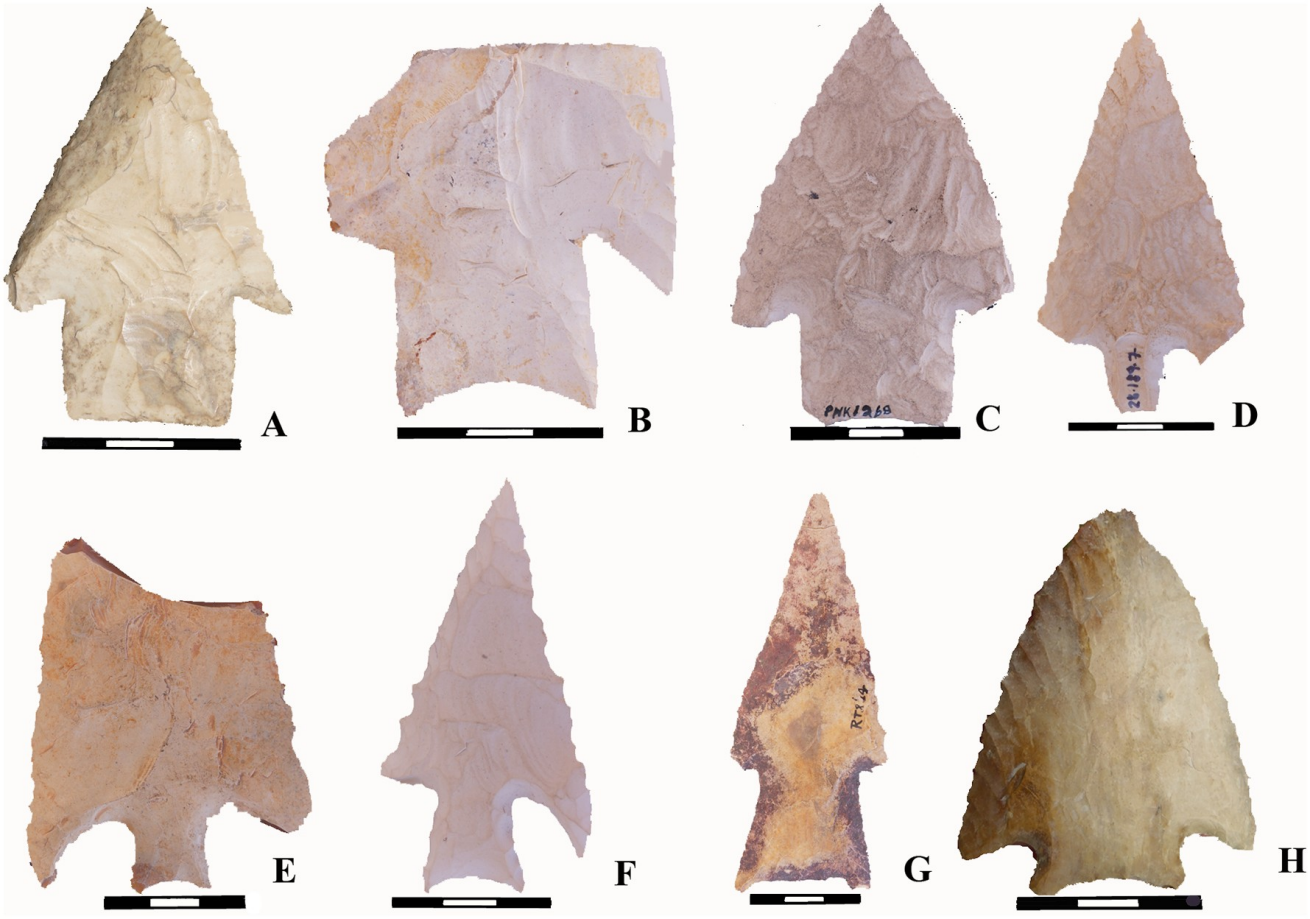
Lowe complex points from Belize. All examples shown here were recovered from surface or undocumented contexts. A-C Lowe type points, D-G Sawmill type points, G Allspice type point, H Ya’axche type point. These examples were photographed with permission of the Belize Institute of Archaeology in 2012.

Since the 1990s Lowe, and a suite of technologically similar bifaces, have become synonymous with the Late Archaic (4,500–3,900 BP). These tools are found exclusively in Belize and are called Lowe, Sawmill, Allspice, and Ya’axche’ types (Fig 2). To date 85 examples have been described in detail [17,26,46,47]. The published frequencies are dominated by Lowe (n = 57) and Sawmill (n = 22), whereas Allspice (n = 4) and Ya’axche’ (n = 2) are poorly represented. These four types likely represent related and contemporaneous, sequential, or overlapping stone tool traditions based on a number of shared characteristics. Commonalities include large points that are stemmed, barbed, and bifacial, with many examples showing unifacial beveling on alternate edges, fluting or longitudinal flake thinning on one side only, concave-to-flat bases, and stem sides that contract from the base to the neck. The overlapping spatial distribution of these points makes it unlikely that they represent products of different cultural groups [46]. Hereafter we refer to this suite of artifacts as *Lowe complex*. They have been recovered from diverse environments ranging from brackish coastal and swampy inland forests in the north, colluvial grasslands and alluvial gallery forests of the Belize River Valley, granitic pine barrens, and upland sandstone foothills and volcanic interior valleys of the southern Maya Mountains. Prior to this study, only seven were recovered during archaeological excavations, and of these only one was found in association with datable organic material.

Though originally described as Paleoindian tools dating to 11,000–8,000 BP [43] and thought to overlap with FPP [45], Lowe points were reassigned as a Late Archaic (4,500–3,900 BP) type in 1993 by Thomas Kelly [17] based on two finds. First, in 1989 in Central Belize two Lowe points were found within ~5 meters of a buried fire hearth, all within a similar orange sandy stratum. The hearth was dated to 3,610 +/- 60, (calBP 4078–3835). However, it was unclear to Kelly whether the orange sand stratum was the result of a single depositional event or if it had been due to water level fluctuations or depositional unconformities, leading him to characterize the association between the date and the artifacts as “weak” evidence. Second, a Lowe point was recovered in excavations of an ancient ditched agricultural field at Pulltrowser Swamp, Belize [48]. Stratigraphically, the Lowe point was recovered from a complex set of soil horizons atop a basal (culturally sterile) clay layer covered by an organic-rich soil and perched 5cm above a peat horizon. The peat unit was described as either the result of fluctuations in the water level or potentially anthropogenic and a “faint paleosol marks the basal clay surface; and a Late Archaic Lowe point, abundant chert debitage, and turtle and fish bones were found just below it. A piece of wood, possibly worked, was associated with the point and was dated to 3,810 +/- 90 radiocarbon years” [49]. As the projectile point was below the paleosol and on the sterile clay layer, it would be difficult to say with certainty that it was contemporaneous with other materials found on that basal surface, compounded by potential disturbances and mixing within contexts directly overlying the Lowe point or from the peat layer, which is stratigraphically below the Lowe point. In the last two decades a great deal of descriptive literature has considered the role of Lowe points in Holocene cultural evolution in southern Mesoamerica [46,50,51], but the chronology has not been revisited despite inconsistencies in the published record and a lack of empirical data.

## Results

We present results of excavations at two rockshelters in southern Belize where we recovered Paleoindian artifacts from well dated stratigraphic contexts. These rockshelters are located 35 km apart along perennial rivers in areas with rich soils and gallery forests. We recovered in stratigraphic context two complete and one partial Lowe complex bifaces and one distal biface fragment with features diagnostic of Lowe complex. In this section we briefly describe the two sites, excavation contexts, and age models.

### Site 1, Tzibte Yux

Tzibte Yux Rockshelter (TY) is a small (37m x 4.5m) rockshelter located 8m above the Rio Blanco and 1.2km from the Classic Maya center Uxbenká [52]. Sediments are a mix of silt, limestone spalls, and dense packed midden (faunal bone from mammals, birds, reptiles, fish, and crabs; lithic debris and expedient flaked tools; charcoal from burnt wood and seeds; and a few scattered human skeletal elements). The upper deposits are dominated by *Pachychilus* sp. (*jute*) snail shells, which are found in abundance in the river below and were culturally modified to facilitate consumption of the snail by removing distal spires [53]. The 110cm deep cultural deposits consist of the *jute* rich midden, which grades into a red clay with decreasing frequencies of *jute* shell. The red clay covers a yellow clay containing few cultural materials directly above limestone bedrock. The upper 30-35cm of the *jute* midden was disturbed in antiquity though there is no indication this was a result of fluvial erosion. Instead this was likely by horizontal removal of the midden during the Classic Period (2,050 to 1,150 BP). Portions of this midden were used as a plaster applied to the back wall of the rockshelter as a frieze or mask containing *jute*, ceramics, lithics, and faunal bone. A single date on wood charcoal embedded in the plaster dates to calBP 10,275–10,190 2*σ* (9,080 +/-35, PSUAMS-1877), indicating preceramic and ceramic-bearing contexts were mixed into the plaster material.

In excavation units 5/6 and 7 the portion of the *jute* midden below the disturbance is highly compacted with sediments in chronological order beginning around 9,000 calBP. Seventeen ^14^C AMS dates (Table 1) document the use of TY during the Late Preclassic through Classic periods (~2,350–1,000 calBP) and from the Paleoindian to the Early Archaic (13,000–8,500 calBP). There is no evidence of Middle or Late Archaic (8,500–3,900 BP) use of the rockshelter.

**Table 1:** Radiocarbon dates used in age models for TY and MHCP. Note, UCIAMS 170149 was not used in the age model given the high error but is included in this table. Depths used in age models were adjusted from depth below datum to depth below surface to account for the uneven and slightly sloping surfaces of both rockshelters. Lab ID are: PSUAMS Pennsylvania State University AMS ^14^C Facility, UCIAMS Keck Carbon Cycle AMS Facility, and DAMS DirectAMS.com. Charcoal samples were prepared at Penn State University and the University of New Mexico Center for Stable Isotopes using standard ABA methods described elsewhere [54]. MHCP chronology is only presented below ~4500 BP.

Site	Unit	Lab ID	Age	error	Depth below surface	Site	Unit	Lab ID	Age	error	Depth below surface
TY	7	DAMS 4078	1885	20	25	MHCP	1	PSUAMS 1197	4485	20	120
TY	7	PSUAMS 1873	2020	20	36	MHCP	1	UCIAMS 151867	4610	25	157
TY	7	UCIAMS 170148	1120	15	47	MHCP	1	PSUAMS 1200	4755	25	159
TY	7	UCIAMS 170155	7910	20	51	MHCP	1	UCIAMS 151871	5075	30	197
TY	7	UCIAMS 170151	8765	25	58	MHCP	1	UCIAMS 142100	5275	25	209
TY	7	UCIAMS 170147	8960	30	59	MHCP	1	PSUAMS 2658	7610	110	214
TY	7	UCIAMS 170160	10060	25	60	MHCP	1	UCIAMS 151870	7775	35	219
TY	7	UCIAMS 170150	10375	25	70	MHCP	1	PSUAMS 2657	9990	45	221
TY	7	UCIAMS 170149*	13850	730	79	MHCP	1	UCIAMS 151872	7940	30	224
TY	7	PSUAMS 2666	13660	70	91	MHCP	1	PSUAMS 2656	8790	45	225
TY	7	UCIAMS 174067	13845	35	91	MHCP	1	PSUAMS 2659	9505	50	230
TY	5	PSUAMS 1874	2010	20	30	MHCP	1	PSUAMS 2660	9450	60	232
TY	5,6	DAMS 4476	8507	35	46	MHCP	1	PSUAMS 2667	9385	45	233
TY	5,6	DAMS 4475	10359	41	48	MHCP	1	PSUAMS 2665	10005	50	255
TY	5,6	UCIAMS 150905	10250	30	48	MHCP	1	UCIAMS 142101	10105	30	257
TY	5,6	UCIAMS 150906	10215	30	53	MHCP	1	PSUAMS 2655	10130	90	259
TY	5,6	UCIAMS 150907	10255	35	59						
TY	5,6	UCIAMS 150908	10355	35	68						
TY	-	PSUAMS 1877*	9080	35	Plaster mask						

* not included in depositional models

**Fig 3:**
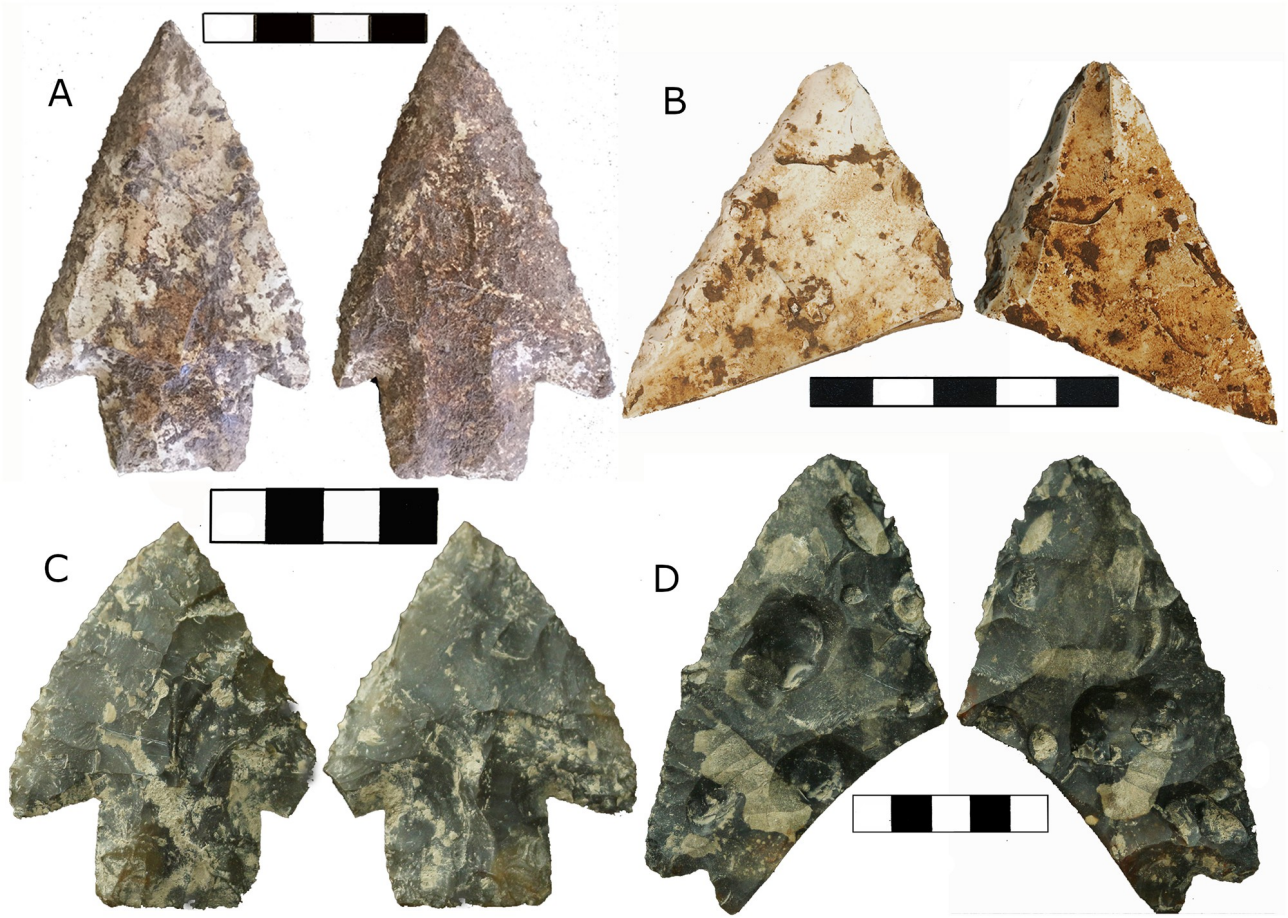
Four Lowe complex artifacts recovered in controlled excavations in southern Belize. (A) Lowe point from TY. (B) Biface distal tip with unifacial beveling on alternate edges from TY. (C) Lowe point from MHCP. (D) Large but badly damaged Lowe point from MHCP. Scales in cm.

**Fig 4:**
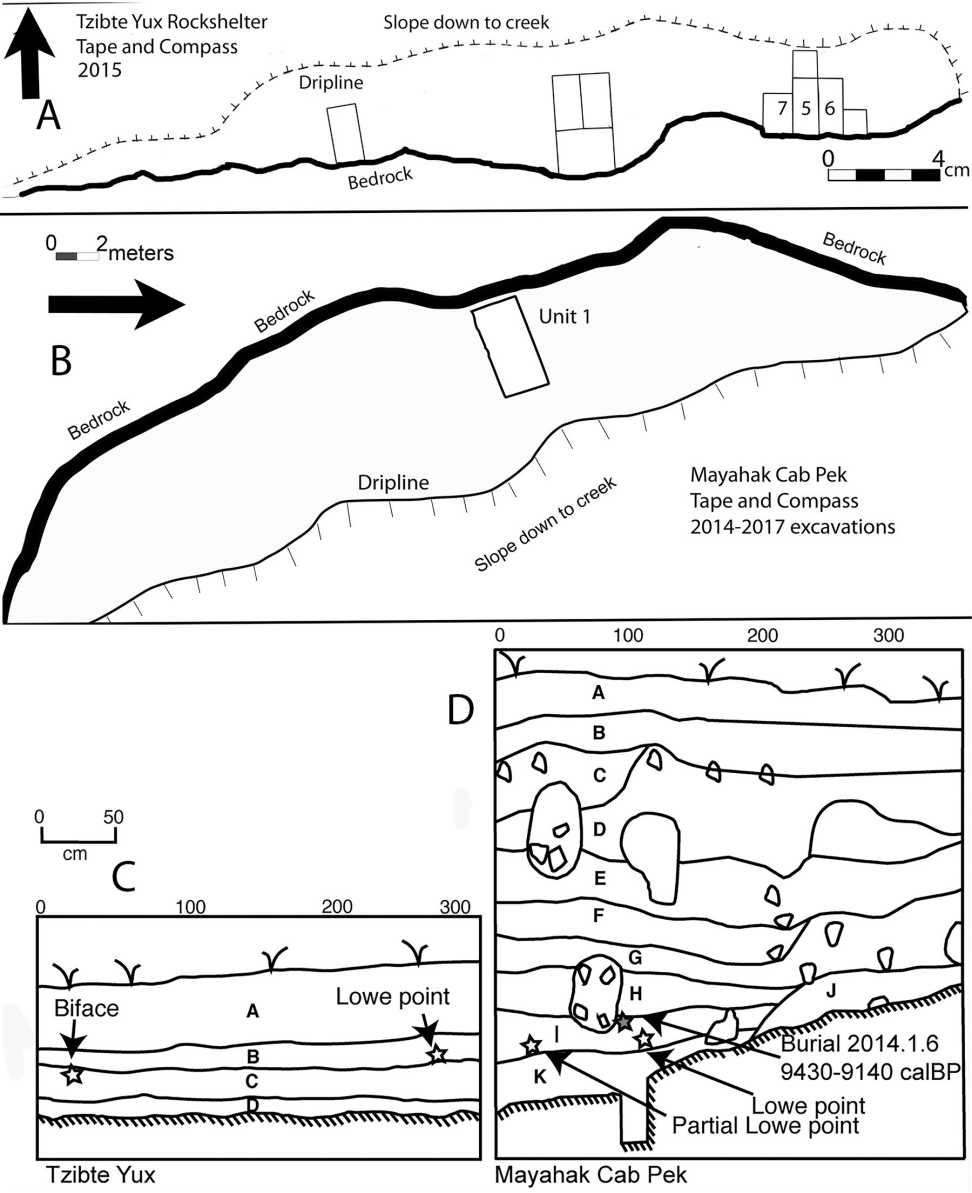
Schematic plans and profiles from TY and MHCP. Plan views (A, B) show locations of excavation units. Number units are those discussed in the text. Profiles (C, D) note locations of Lowe bifaces discussed in the text and major stratigraphic units. At TY (C) horizons are A: unconsolidated *jute* midden, B: consolidated *jute* middle, C: red clay layer, and D: yellow clay layer. At MHCP (D) major horizons A-D alternating organic rich rocky middens and dense *jute* lenses while G-K are silty middens with decreasing *jute*.

A Lowe point (Fig 3a) was recovered from the boundary between the undisturbed midden and the red clay in Unit 7 (Fig 4a and 4c). The stemmed point is barbed, has unifacial beveling on alternate edges, and a 24mm flake scar strikingly similar to a flute on one side of the stem. Length, width, thickness, neck width, and tip cross-sectional area (TCSA) are all within 1 SD of other published Lowe points (Table 2). The point was within 1cm of wood charcoal that dated to calBP 10,223–9,929, 2*σ* (8,960 +/-30, UCIAMS-170147). This is one of a sequence of five dates directly above and below the point ranging from calBP 8,949–8,607, 2*σ* (7,910 +/-20, UCIAMS-170155) to calBP 12,391–12,087 2*σ* (10,375+/-25 UCIAMS-170150). In units 5/6 we recovered a distal end fragment of a large biface (Fig 3b) with steep unifacial beveling on alternate edges from the red clay layer in direct association with charcoal dating to calBP 12,399–12,034 2*σ* (10,359+/-41, DAMS-4475). This date is also part of a series of five sequential assays above and below the biface.

**Table 2:** Lowe type point metrics for TY and MCHP complete examples compared to previously published [46] examples for Length (L), Width (W), Thickness (T), Neck Width (NW) and tip cross-sectional area (TCSA).

Data	Mean L [mm]–(N)	Range L [mm]	SD	Mean W [mm] (N)	Range W [mm]	SD	Mean T [mm]–(N)	Range T [mm]	SD	Mean NW [mm]–(N)	Range NW [mm]	Mean TCSA [mm^2^]–(N)	Range TCSA [mm^2^]	SD
**Previously publsihed Lowe Points**	**83.7 (33)**	**59.3–139**	**18.6**	**55.3 (35)**	**43.6–78.0**	**6.7**	**9.8 (20)**	**6.0–12.1**	**1.3**	**29.1 (42)**	**21.2–39.4**	**257.7 (16)**	**130.8–332.8**	**48.2**
TY Lowe Point	78.7			48.6			9.6			25.2		233.3		
MHCP Lowe Point	68.9			54.6			11.1			28.4		303.3		

**Fig 5:**
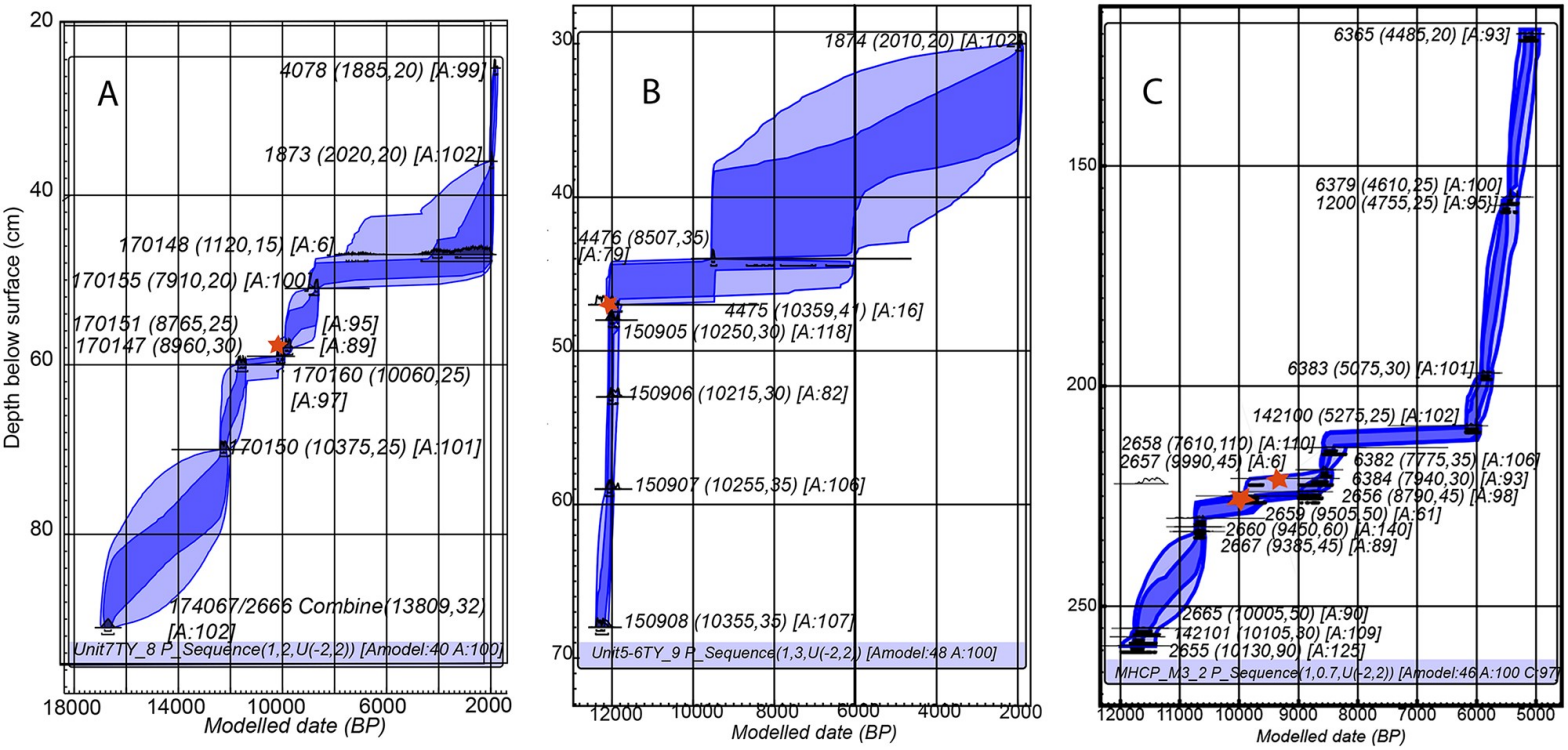
**Poisson depositional models for (A) TY Unit 7. (B) TY Unit 5/6, and (C) MHCP Unit 1** Stratigraphic and temporal locations of Lowe complex artifacts are indicated by red stars (see text for detail on each context). Each modeled date consists of lab-ID (radiocarbon age, error) [A = model agreement]. Dark blue shading is 1σ model error and light blue shading is 2σ model error. Model data are in Table 1.

To test the integrity of our chronology we developed depositional models for both units at TY (Fig 5a and 5b). These consistently show poor model agreement and high 1*σ* and 2*σ* model errors post-8,500 calBP with age reversals in the disturbed *jute* midden, but excellent agreement and low 1*σ* and 2*σ* model errors in the consolidated lower levels of the *jute* midden and red and yellow clay layers. The basal age of Unit 7 is very early, calBP 16,939–16,474, 2*σ* (model combined 2 dates 13,660+/-70, PSUAMS 2666 and 13,845+/-35 UCIAMS 174067). A third concordant date on a carbonized seed from 10cm above (13,850+/-730, UCIAMS-170149) was not modeled because of its larger error due to small sample size but is included in Table 1. The context where the seeds and charcoal were recovered also included small amounts of flaked chert debitage and fragments of faunal bone of small unidentifiable mammals. While these dates are consistent with some very early pre-Clovis contexts [55,56] we consider any association of this date and human activity to be provisional and pending additional excavation and documentation.

### Site 2, Mayahak Cab Pek

Mayahak Cab Pek (MHCP) rockshelter is located in an interior valley of the Maya Mountains in the Bladen Nature Reserve, a protected wilderness area where there has been minimal modern human disturbance of archaeological sites. Excavations of dry midden rich stratified deposits show that cultural use began prior to 12,000 BP and continued through the Maya Classic Period, until 1,000 BP. The shelter is east-facing with a 20m high limestone face containing caramel-colored chert lenses and cobbles. The shelter is approximately 20m above the present-day stream bed of an unnamed tributary of the Bladen Branch of the Monkey River and has cultural deposits over 3m deep.

MHCP Unit 1 (Fig 4b and 4d) was a 2.5 x 2.5m excavation conducted over three field seasons (2014, 2016–17) and ending at a depth of 280cm. Excavations were conducted based on 5-10cm levels and observed stratigraphic changes. The ceramic bearing upper portions of the stratigraphy (Fig 4b, strata A-D) can be generally characterized as repeating two sub-stratigraphic soil units, which include midden fill overlying concentrations of cobbles that likely represent occupation surfaces. The midden deposits contain abundant flaked stone (both chert and igneous rock), faunal remains of mammals, birds, and reptiles, and ceramics, much of which shows evidence of burning. Spire lopped *jute* shell concentrations comprise up to 50 percent of the matrix.

The aceramic levels are organic rich black silt to silty-loam fine grained matrix with cobble clasts of limestone mixed with crude porphyritic igneous flaked tools, mostly choppers and hammer stones with battered edges. Artifact and faunal concentrations are moderate and *jute* concentrations also drop well below 30% of matrix except for at the ceramic-aceramic boundary, where they are the most abundant of any period. The overall size and the number of cobbles, expedient tools, and faunal remains tend to increase with depth. In levels below 9,000 BP sediments became increasingly silty. Within the silty matrix we found a smaller frequency of large chert flakes and crude bifaces. These silty matrices terminate on boulders intermixed with reddish clays and decaying limestone devoid of any cultural materials.

Contexts with human remains from at least fourteen individuals have been recovered from Unit 1 and range in date between 1,000 and 10,500 BP. Although the analysis of these remains is not reported here burial MHCP.14.1.6, the disarticulated skeleton of a female, directly was dated on bone collagen to 9,300 BP [1]. Genetically, she belongs to haplogroup D4h3a, linking her directly to the Anzick child skeleton from Montana [57] who is considered an ancestor of founding North, Central, and South American populations [58].

One complete and one partial Lowe point (Fig 3c and 3d) were recovered from MHCP Unit 1. The complete point has one well-defined barb, unifacial beveling on alternate edges, and two longitudinal thinning flake scars on one side of the stem with the longest being 23mm. Length, width, thickness, stem width and TCSA are within 1σ of other known Lowe points (Table 2). The other point is badly damaged, missing the entire stem, both barbs, and the distal tip, and has battered edges as well as fire damage. The blade portion of this biface is in early stages of rejuvenation with thin edges, which are opposite-edge flaked but show no bevel. Both points were recovered from the same stratigraphic level, containing lithic, faunal, and isolated human remains. Two charcoal samples were found directly below the complete point, one 5cm lower dating to calBP 10,131–9,606, 2*σ* (8,790+/-45, PSUAMS-2656) and one 3cm below, although out of sequence and treated as a model outlier, dating to calBP 11,701–11,266, 2*σ* (9,990+/-45, PSUAMS-2657). Conservatively, we could associate the minimum age of the point with the younger of these two dates. The partial Lowe biface was found 90cm to the east of the complete point, in the same stratigraphic level but not in direct association with any datable materials. Charcoal from 2cm below and 66cm south of the biface dates to calBP 8,609–8,451 (7,775 +/-35, UCIAMS-151870) but is likely intrusive or dating a context that is younger than the biface based on the slope of the bedrock below and its location in the sequence of dates. Both points were found below the burial 2014.1.6 [1], which is directly dated on purified bone collagen (9,430–9,140 calBP (combined UCIAMS-151854; UCIAMS-151855). This suggests that the minimal age for the Lowe bifaces should be older than the burial.

The depositional model of MHCP Unit 1 (Fig 5c, Table 1) uses 16 radiocarbon dates from charcoal recovered from matrix level fill from pre-ceramic levels. We excluded dates from within feature pits (fires and burials) because they tended to be slightly younger than the surrounding matrix, including Burial 2014.1.6. Two dates from MHCP Unit 1 were rejected based on unresolved depth issues and clear chronological inconsistency. These rejected samples were from ~4,000 calBP levels not near to the Lowe points. Overall, model agreement is high and 1*σ* and 2*σ* errors low for all age-depth samples (>85%), with the exception of one outlier mentioned above.

### Methods: AMS Dating and Bayesian depositional modeling

All our field research, exportation of archaeology samples, and destructive analysis of charcoal samples was done with permits from the Belize Institute of Archaeology (IA/H/2/19(06)), and the Belize Forest Department (FD/WL/1/19(05)) issued to KMP.

AMS dates on charcoal were prepared using published Acid-Base-Acid, combustion, and graphitization methods at the Pennsylvania State University (PSU), University of California at Irvine (UCI), and University of New Mexico [59] and measured on NEC Accelerator Mass Spectrometers at PSU and UCI. Three TY samples were processed and analyzed at Directams.com using similar protocols available on their website. Samples selected for this study were all from point-plotted (X,Y,Z) locations and recovered from unit-fill rather than within intrusive features, such as burials, which tend to not reflect overall depositional processes. Preference was given in sample selection for short lived seeds or twigs when possible.

Age models were developed using stratigraphic data from TY and MHCP with depths adjusted from cm below datum to cm below surface to account for surface and basal slope in excavation units. Bayesian Poisson depth models were produced in OXCAL 4.3 [60] using published parameters [15,16]. In this method a variable k defines the depth of deposition events and the prior is expressed as: v = log_10_/ (k/k_0_) which allows for depositional variability over a wide range [61]. It is shown as:

$$\text{k}=k_{0}10^{v}$$

v=~D

Where D is a prior distribution expressed over a reasonable range. In Oxcal we use a depositional P_Sequence command (P_Sequence(name,k_0_,p,D)) where *k*_0_ is the base *k* parameter, *p* is the interpolation rate, and *D* is the prior distribution for *v*, and the sedimentary sequence defined in terms of depth in cm. For example, P_Sequence(1,2,U(-2,2)) defines *k*_*0*_ = 1cm^–1^, interpolation rate = 2 cm^–1^ (output every 5 mm), and k allowed between a factor of 10^−2^ and 10^2^.

Chronologies from both rockshelters bracket the minimum age of the TY and MHCP Lowe points as calBP 10,223 and 9,300 2*σ*, and the deposition of the technologically related unifacially beveled biface fragment from TY minimally as calBP 12,399–12,034 2*σ*. There is good agreement in Poisson depositional models for all excavation contexts containing Lowe complex tools with dates on both complete Lowe points at ~10,000 calBP.

## Discussion

At the end of the Pleistocene, bifacial stone tool technologies were widespread across the New World [28,62]. By 12,700 BP Clovis was no longer manufactured [31] and Lowe complex bifaces likely appear towards the end of the FPP traditions and the NA Folsom tradition. Both early technocomplexes are spread over large subcontinental areas [35,63,64], but Folsom is restricted to the Great Plains and western NA and has not been reported for the neotropics. FPP have primarily been recovered from SA, from the Southern Cone to the Amazon, but are also found as far north as southern Mexico [65,66]. Subsequent late Paleoindian traditions have greater diversity in biface types reflecting increasing regionalization in NA [67] and SA [38–40,68]. We suggest that Lowe complex points (Lowe, Sawmill, Allspice, and Ya’axche’) represent such a regional lithic tradition with distinctive features that are shared with some technological complexes in NA and others in SA, but with a primary focus on tropical regions of lower CA and northern SA.

The most distinctive NA technological feature in the Lowe complex is unifacial beveling on alternate edges. This appears first in the Folsom ultrathin bifacial knives as a sharpening technique with steep edge angles, centered on the Great Plains but extending southward into Chihuahua, Mexico from 12,610–12,170 calBP [36,69]. This overlaps with the earliest example of unifacial beveling on alternate edges at TY. There is no evidence of unifacial beveling on alternate edges on FPP or any other SA tool types, nor on any of the earlier lanceolate or other Clovis bifaces found in CA.

Following Folsom, but in the southeastern US, Dalton (10,400–9,850 BP, though poorly dated) may be the first projectile points with unifacial beveling on alternate edges [70]. It is also found on younger (9,800–8,000 BP) square-stemmed Cody knives [71] and Foothill-Mountain Tradition Pryor Stemmed points [72]. In the Midwestern US Hardin Barbed bifaces bear striking morphological resemblance to Lowe points, exhibiting square to slightly expanding stems with blades that may exhibit projecting barbs where they meet the stem. Steep unifacial beveling is seen on some examples [20], which are thought to be younger than Dalton.

Experimentation with Dalton replicas suggest that beveling may promote rotation and accuracy of thrown spears [73] when large beveled points are hafted on short inflexible shafts. Other experiments have found the opposite, that beveling is not directly associated with unidirectional rotation, but have proposed they might rotate in the flesh of an animal on impact [74], inflicting greater damage. However, it seems unlikely in a tropical forest one would be regularly throwing a spear with a hafted Lowe point by hand in a manner that would generate the velocity needed to spin it significantly, effectively nullifying the benefits of either rotation, aerodynamics, or deep wounding. In contrast, beveling of older Folsom ultrathin and Cody knives appears to have been a resharpening technique for cutting tools [75] that could be equally effective on thrusting harpoons or spears. Unifacial beveling on alternate edges is present on all Lowe complex types, although not on all examples, and Lowe complex types are too thick (mean 9.8mm, Table 2) to have been tips for arrows or, with the exception of some thinner Sawmill types, atlatl darts [46]. This blade rejuvenation technique has been argued as evidence of Lowe bifaces being fashioned as hafted knives, harpoons, or thrusting weapon tips [17]. It is possible that these represent a continuum from large early stage knives to end reduction stage projectiles with shifting functions [76].

Basal thinning or fluting is both a NA and SA Paleoindian technology, but fluting or thinning on only one side of a biface appears to be limited to CA and SA. NA Clovis and Folsom are distinctly fluted, as are many Dalton points [20], with single channel flakes removed from both sides of the base. In NA single side fluting is considered unusual [77]. with the exception of Northumberland points from the eastern U.S. [78] and possibly some far western Clovis points [79].

Single side thinning or fluting is associated with Lowe complex and with Paleoindian points in lower CA and SA. Both fluting and longitudinal thinning flake removal are observed on FPP and El Inga points [80], frequently on one side only [81–83]. In one SA study 60.2% of fluted FPP are single side fluted or thinned [84]. At El Gigante in western Honduras, seven complete or partial bifacial projectile points were recovered from Paleoindian contexts dating to 10,000–9,100 BP [23]. Several are described as having “opportunistic vertical thinning flakes taken from one side of the stem base” [85]. These points are very similar to examples of basal thinning on end of use life points from Pay Paso, Uruguay, a SA late Paleoindian site dating to 11,000–9,000 BP [65]. Fluted points from sites in highland Guatemala [25,30], Northern Belize [86] and Costa Rica [87] are illustrated having single sided fluting, sometimes with possible minimal thinning on the reverse. At Turrialba, Costa Rica [88], both Clovis and FPP have been recovered from surface collections and test pits, two of which are single side fluted while others have flutes and thinning flakes on both sides.

Barbed bifaces, some of which have the square stems and unifacial beveling on alternate edges, are characteristic of the Lowe complex. In NA barbed bifaces do not appear until just before 10,000 BP [20]. In SA triangular stemmed barbed points are earlier, first found in the Tigre complex in Uruguay and southern Brazil by 12,000 BP [40], the Punta Negra points in the Atacama Desert by 12,000 BP [8], in the Paijan complex dated to around 13,000–11,000 BP in the northern coast of Peru [38,68,89], and in the Amazon at least by 11,000 BP [90]. As these cases suggest, these points often replace FPP implying some form of interregional consistent stylistic and technological transition. Barbed bifaces have also been found in undated but likely early Holocene contexts in lower CA including at Madden Lake, Panama [44], and unusual fluted and stemmed varieties from the Curua Basin, Brazil [91], as well as the Rio Magdalena and Region del Jobo, Venezuela [92] and Pay Paso, Uruguay [93]. The occurrence and dating of barbed bifaces place this technocomplex as an earlier phenomenon in SA than in NA and suggests a possible source for the technological knowledge related to Lowe complex bifaces.

The suite of traits present on Lowe complex points suggests that they served several functions. Barbs are characteristic of penetrating spears but can also be used as hooks, a handy tool in a rainforest to harvest or cut palm fruits or vines. Unifacial beveling on alternate edges with minimal thinning is one way to maintain a durable cutting edge, but on thin aerodynamic projectiles may increase accuracy. Function was likely dependent on size, thickness, and degree of rejuvenation and reduction, and during the use-life of a biface it may have served multiple functions. Further, in broadleaf forests of the early Holocene some projectiles may have been less effective as weapons given the density of trees and foliage.

### Population structure, gene flow, and technological diffusion

Transmission of Paleoindian stone tool techno-knowledge from NA to CA and SA has been proposed and discussed [82] but can now be informed by paleogenetic studies which demonstrate the timing and structure of waves of humans migrating from NA into and through the bottleneck of CA, then colonizing SA [1,58]. Early colonizers likely brought distinctive NA stone tool technologies with them. We also see continuity between barbed Lowe complex points and late Paleoindian tools from lower CA and SA.

Genetic data reveal that all Native Americans derived from a single homogeneous ancestral population that diversified sometime between 16,000–13,500 BP, likely south of the NA ice sheets [94]. The resulting ancestral branches are described as “*Southern Native American*” or (ANC-A) and “*Northern Native American*” (ANC-B) [57,95,96]. The ANC-B branch is closely linked to Native American populations from eastern NA and the ancient Alaskan skeleton USR1 (11,600–11,270 calBP) [58]. The ANC-A branch was the source of at least four population movements from NA to CA and SA prior to 7,000 BP, including a 9,300-year-old skeleton from MHCP, two 7,400-year-old skeletons from Saki Tzul (located 1.2 km from MHCP), and five newly reported ancient genomes from Chile, Brazil, Peru, and Argentina dating between 10,900 and 7,000 BP [1]. These genetic data indicate that ancient individuals from southern Belize prior to 7,400 BP likely derive from western Clovis ancestors and are closely related to ancient and modern SA and lower CA populations with less allele sharing with modern indigenous populations in central or northern Mexico. Our earliest Bladen populations were the descendants of ANC-A populations dispersing across tropical CA and into SA. They may share alleles with the people who developed FPP in SA prior to ca.12,000 BP [35]. These data are consistent with a hypothesis that most of the sharing of stemmed tool technologies during the late Paleoindian period between populations in Belize was with their neighbors to the south, in lower CA and SA, and less sharing between Belize and populations in NA and central/northern Mexico. It remains possible that Pleistocene coastal travelers brought stemmed points to SA [97] before the start of FPP, but this is hard to reconcile with the fact that stemmed traditions do not appear in NA until after they were well established in SA. Unifacial beveling on alternate edges appear simultaneously in NA and CA around 12,200 BP suggesting some transmission of knowledge related to this technological feature but more data from both stratified sites with bifacial stone tools and ancient DNA are needed to fully test these propositions.

## Conclusions

The Lowe tradition is a technological lithic complex unique to southern Mesoamerica. It shares features with contemporaneous stemmed point types found primarily in tropical areas of SA and lower CA, but is also related to ancestral Paleoindian complexes in NA. Its development corresponds with a pattern of regionalization and diversification reflected in similar age tools found throughout the Americas [98] at a time of changing Holocene environments. Links to NA are complicated by a dearth of supporting data on early technocomplexes from the large geographic space encompassed by most of central and southern Mexico, making it hard to find evidence for lateral cultural transmission of technological knowledge from NA to southern Mesoamerica.

Lowe complex tools are basally thinned, stemmed, and barbed bifaces. They are a flexible tool type well suited for the diverse set of early Holocene environments like those that emerged in Belize. In particular, tropical forests offered new resources that were of economic importance including a wide variety of palms, tubers, and vines [99,100]. Broad-spectrum plant and animal-based economies were likely well in place in the late Paleoindian period in CA [101], much like they were in SA [62,102]. Comparative data from Chiapas in southern Mexico suggest flaked stone tools of late Paleoindian age were used for plant and wood processing [103] similar to proposed diversified plant-based economies in lower CA [101,104] and SA [105].

We provide a chronology for the Lowe complex that is potentially 2700 years long, far exceeding any existing New World Paleoindian technocomplex. Unifacial beveling on alternate edges, not found on other South or Central American points and not found on earlier Clovis or lanceolate types, may be the earliest characteristic of Lowe complex. Our biface tip from TY dates to calBP 12,399–12,034 and is steeply beveled. Our Lowe point type bifaces (n = 3) all date between calBP 10,223 and 9,300, indicating they are Late Paleoindian in age. This suggests that Lowe type bifaces were manufactured over a shorter time-period, while the overall complex that includes opposite edge beveling technology of large bifaces in Central America may have lasted significantly longer, though it is less clearly defined. In this paper we argue that the Late Paleoindian period is a time of diversification in tool types as an adaptation to the range of environmental conditions found in southern Mesoamerica as well as increasing reliance on plant resources, and that this specialization is a feature you see very early on in the occupation of the neotropics, and likely in adjacent regions of South America.

We lack any chronological data for Sawmill, Allspice, or Ya’axche type points which we include in the Lowe Complex based on shared characteristics we describe above. We would note the similarities between both Sawmill and Ya’axche type points and some examples of stemmed el Jobo from Venezuela [106], and el Inga [82] from Ecuador. Future research needs to determine temporal relationships between Sawmill, Allspice, and Ya’axche types, continue to refine the chronology of alternate edge beveling technology, and add to the number of overall AMS dates from stratified paleoindian sites. Further, our understanding of Paleoindian subsistence will be enhanced with more detailed analyses of other stone tools from these assemblages, including grinding, chopping, and cutting tools made of non-chert resources. These studies accompanied by macrobotanical and residue analyses will clarify the range of subsistence resources exploited by early settlers.

This reappraisal of the chronology of Lowe complex fills an important gap in the prehistory of southern Mesoamerica, a particularly diverse resource rich tropical landscape. It is also the first securely dated Paleoindian tool for this region. Shifting the chronology of the Lowe complex from 4,500 BP to as early as 12,000 BP leaves a notable temporal gap with no bifacial technocomplex for CA for the Early to Late Archaic (9,000–3,900 BP). Middle/Late Archaic bifacial technocomplexes are notably absent from the well dated sites across lower CA and SA. In southern Belize formal bifacial tools are not present in Archaic assemblages, as is the case for El Gigante [85]. This is again in stark contrast to NA with its robust Archaic technocomplexes. Southern Mesoamerica again looks a lot more like its tropical neighbors to the south [107], suggesting little lateral technological transmission from NA to the tropics across this time period.
